# Diagnostic Predictive Scores of Amyloid Cardiomyopathy in Patients with Heart Failure with Preserved Ejection Fraction and Left Ventricular Hypertrophy

**DOI:** 10.3390/jcdd12110417

**Published:** 2025-10-22

**Authors:** Denise Cristiana Faro, Fabrizia Romeo, Valentina Losi, Dario Simonetti, Davide Capodanno, Ines Paola Monte

**Affiliations:** 1 Department of General Surgery and Medical-Surgical Specialties, University of Catania, Via S. Sofia 78, 95123 Catania, Italy; fabriziaromeo97@gmail.com (F.R.); vale.losi@gmail.com (V.L.); dario.simonetti92@gmail.com (D.S.); dcapodanno@unict.it (D.C.); 2 Cardiology Unit—CAST, AOU Policlinico “G. Rodolico-S. Marco”, Via S. Sofia 76, 95123 Catania, Italy

**Keywords:** diastolic dysfunction, amyloidosis, HFpEF, restrictive cardiomyopathy, Echocardiography, diagnostic scores

## Abstract

Background: Wild-type transthyretin cardiac amyloidosis (ATTRwt-CM) is a frequent but underdiagnosed cause of heart failure with preserved ejection fraction (HFpEF) and left ventricular hypertrophy (LVH). Early identification is essential given the availability of disease-modifying therapies. The T-Amylo and Davies scores are non-invasive tools for estimating ATTR CM probability, but their comparative performance in the same real-world population is not well defined. Objectives: To compare the diagnostic accuracy of T-Amylo and Davies scores in consecutive patients referred for suspected cardiac amyloidosis. Methods: We retrospectively analyzed 81 patients (mean age 76.8 ± 8.3 years, 74% male) who underwent a standardized work-up: ECG, echocardiography with strain, NT-proBNP and troponin, bone scintigraphy, and immunofixation. ATTR CM was diagnosed according to established non-biopsy criteria. Both scores were calculated retrospectively, and sensitivity, specificity, predictive values, accuracy, and agreement were assessed. Results: ATTR CM was confirmed in 28 patients (34.5%). T-Amylo showed higher sensitivity (91.2% vs. 73.5%) and NPV (89.7% vs. 79.1%), while Davies had greater specificity (85.0% vs. 65.0%) and PPV (80.5% vs. 70.8%). Overall accuracy was comparable (T-Amylo 77.0% vs. Davies 79.7%). Agreement between scores was moderate (κ = 0.59). Conclusions: T-Amylo is best suited as a screening tool for suspected ATTR CM, while Davies offers confirmatory value in high-probability cases. Combining these tools in a sequential strategy may optimize diagnostic efficiency, reduce unnecessary testing, and expedite initiation of disease-modifying therapy.

## 1. Introduction

Cardiac amyloidosis (CA) has emerged as a significant and underrecognized cause of heart failure with preserved ejection fraction (HFpEF) and unexplained left ventricular hypertrophy (LVH), especially in older adults [1,2,3]. Recent population-based screening using DPD scintigraphy in undifferentiated HFpEF patients aged ≥ 60 years revealed a prevalence of wild-type transthyretin amyloidosis (ATTRwt-CM) of approximately 8%, with affected individuals more often being older males with increased LV wall thickness and elevated troponin [4]. Another large Spanish multicenter study reported a prevalence of 16.8% among HFpEF patients with LVH ≥ 12 mm [5]. These findings underscore the importance of systematic screening for ATTRwt in elderly HFpEF cohorts.

ATTRwt results from age-related misfolding of transthyretin (TTR), leading to fibril deposition in the myocardium, diastolic dysfunction, and eventual restrictive physiology [1,6]. Although TTR genetic variants (ATTRv) may mimic this presentation, ATTRwt predominates in patients older than 60–70 and is often associated with extracardiac red flags such as bilateral carpal tunnel syndrome and lumbar spinal stenosis [7,8].

Distinguishing ATTRwt from monoclonal immunoglobulin light-chain amyloidosis (AL-CM) is critical, as AL-CM requires prompt hematologic therapy due to its rapid progression and poorer prognosis. Current criteria recommend suspicion of CA in patients aged ≥ 65 years with LVH ≥ 12 mm in the absence of significant hypertension or valvular disease [1,9].

The advent of non-invasive diagnostic modalities, particularly 99mTc-DPD/PYP/HMDP bone scintigraphy, has facilitated ATTR-CM diagnosis without the need for biopsy, provided monoclonal protein is excluded via serum/urine immunofixation and free light chain assay [10]. Early identification is vital given the availability of disease-modifying therapies such as tafamidis and acoramidis and emerging gene-silencing agents (patisiran, inotersen, vutrisiran), together with the monoclonal antibody-mediated removal of ATTR deposits and genetic knockdown of TTR expression via the CRISPR-Cas9 system currently being tested in phase 2–3 clinical trials [11,12,13,14,15,16,17].

To streamline screening for ATTR-CM, clinical prediction tools have been developed. The T-Amylo score, derived and validated in a large multicenter cohort by Arana-Achaga et al. (2023), incorporates age > 65, LV wall thickness ≥ 12 mm, apical sparing on strain, ECG abnormalities (low-voltage/pseudo-infarct), elevated NT proBNP or troponin, and bilateral carpal tunnel syndrome, demonstrating strong discrimination (AUC ≈ 0.86) [18]. The Davies score, published in JAMA Cardiology (2022), is a simpler six-variable model including LVH, NT-proBNP > 3000 pg/mL, low QRS voltage, and absence of monoclonal protein, validated in HFpEF cohorts with high specificity (~85%) [19].

While both scores have proven useful in different contexts, no prior study has directly compared their diagnostic performance head-to-head in a real-world population undergoing standardized, multimodal evaluation for suspected CA. Understanding how these tools perform in the same cohort—and their level of concordance—is helpful for optimizing diagnostic pathways, especially in settings where access to advanced imaging may be limited.

The aim of the present study was to compare the diagnostic performance of the T-Amylo and Davies scores in a consecutive series of patients referred for HFpEF with presence of LVH and suspected cardiac amyloidosis. All participants underwent a comprehensive evaluation including echocardiography with strain, ECG, biomarkers, bone scintigraphy, and immunofixation. We sought to determine and compare the sensitivity, specificity, predictive values, and overall accuracy of each score, and assess their diagnostic concordance in the same patient population.

## 2. Materials and Methods

### 2.1. Study Design and Population

We conducted a retrospective observational study including consecutive adult patients referred to our tertiary referral center between January 2021 and June 2023 presenting with unexplained left ventricular hypertrophy (LVH) and heart failure symptoms, for evaluation of suspected cardiac amyloidosis (CA). The study methods followed the STROBE Checklist for observational studies (Supplemental Material S1).

Patients were included if they fulfilled all the following criteria:

1. Age ≥ 18 years;
2. LV wall thickness ≥ 12 mm on transthoracic echocardiography (TTE) and evidence of preserved ejection fraction (>50%);
3. Signs and symptoms of heart failure;
4. Availability of a complete diagnostic work-up including
  - A 12-lead electrocardiogram (ECG);
  - Cardiac biomarkers [N-terminal pro–B-type natriuretic peptide (NT-proBNP) and high-sensitivity troponin];
  - TTE with longitudinal strain analysis;
  - Bone scintigraphy with 99mTc-DPD or 99mTc-HMDP;
  - Serum and urine immunofixation and free light chain (FLC) assay.

Patients with incomplete data, lacking a suitable acoustic window for the acquisition and analysis of good-quality echocardiographic images, not collaborative, with a previously confirmed CA diagnosis, or having undergone prior treatment with disease-modifying therapy (e.g., tafamidis, patisiran, inotersen) were excluded.

### 2.2. Diagnostic Work-Up and Reference Standard

All patients underwent a standardized diagnostic protocol aligned with current international recommendations [1,9]. ATTR-CM diagnosis was established non-invasively when the following were present:

- Grade 2 or 3 myocardial uptake on bone scintigraphy;
- Absence of monoclonal protein on immunofixation;
- Normal FLC ratio.

In cases with Grade 1 uptake or inconclusive results, endomyocardial or extracardiac biopsy was performed when clinically indicated. Patients were classified into

- ATTR-positive: confirmed ATTR-CM (wild-type or variant);
- ATTR-negative: no evidence of ATTR-CM (including AL-CM and other LVH causes).

#### 2.2.1. Electrocardiography and Echocardiography

Standard 12-lead ECG recordings were assessed for rhythm, conduction abnormalities, QRS voltage (Sokolow–Lyon index), and pseudo-infarct pattern. Low QRS voltage was defined as a QRS amplitude < 5 mm in all limb leads or <10 mm in all precordial leads, according to established criteria [20,21].

TTE was performed using a Vivid E95 system (GE Healthcare, Oslo, Norway) by experienced sonographers blinded to clinical data. Analyses were conducted offline using EchoPAC v2.02 (GE Healthcare, Chicago, IL, USA). Measurements included interventricular septum and posterior wall thickness, left ventricular mass index (LVMi), left ventricular ejection fraction (LVEF), diastolic parameters (E/A ratio, E/e′), left atrial volume (LAVi), maximal velocity of tricuspical regurgitation (TRVmax), presence and severity of valve diseases (mitral regurgitation or aortic stenosis), and pericardial effusion. Global longitudinal strain (GLS) was derived via speckle-tracking analysis. An apical sparing pattern was identified from the strain bull’s-eye plot, defined by a relative preservation of apical strain compared to basal and mid segments (apical sparing was primarily identified visually, and in doubtful cases, the relative regional strain ratio and septal apical-to-basal strain ratio were calculated, with values > 1 and >2.1, respectively, considered consistent with apical sparing, in line with previously published methodology) [22].

#### 2.2.2. Bone Scintigraphy and Immunological Testing

All patients underwent planar and SPECT imaging 3 h after the injection of 99mTc-DPD or 99mTc-HMDP. Myocardial uptake was graded using the Perugini score (0–3) by two nuclear medicine physicians blinded to other test results.

Serum and urine immunofixation and serum free light chain assays were performed in all cases. A κ/λ FLC ratio outside the range 0.26–1.65 was considered abnormal.

#### 2.2.3. Scoring Systems Evaluated

Two scores were applied to all patients (Table 1):

- T-Amylo Score:

Age > 80 years, LVH (IVSd ≥ 16 mm), male gender, ECG abnormalities (low voltage), carpal tunnel syndrome. Threshold for high probability: score ≥ 7 points.

- ATTR-CM (Davies) Score:

Age, male gender, ejection fraction < 60%, LVH (posterior wall thickness ≥ 12 mm, relative wall thickness > 0.57), and a previous hypertension diagnosis (the last one scored minus 1 point). Score ≥ 6 is considered high-risk for ATTR-CM.

### 2.3. Statistical Analysis

Continuous variables are presented as means ± standard deviations or medians [interquartile ranges] depending on distribution (Shapiro–Wilk test). Categorical variables are expressed as counts and percentages.

Between-group comparisons used Student’s t-test or the Mann–Whitney U test for continuous data, and the chi-square or Fisher’s exact test for categorical data.

Diagnostic accuracy was assessed by sensitivity, specificity, positive predictive value (PPV), negative predictive value (NPV), and overall accuracy using confirmed ATTR-CM as the reference.

Agreement between scores was evaluated using Cohen’s kappa statistic.

ROC curves were generated for each score, and area under the curve (AUC) values were compared using the DeLong method.

Statistical analyses were performed with SPSS Statistics ver 27.0 (IBM Corp.). Two-sided *p*-values < 0.05 were considered significant.

### 2.4. Ethical Considerations

The study was conducted in accordance with the Declaration of Helsinki and Good Clinical Practice. Informed consent was obtained from all subjects involved in the study at the time of the first visit for the anonymous and aggregated use of their clinical data for research purposes.

## 3. Results

### 3.1. General Characteristics of Study Population

A total of 81 patients met the inclusion criteria (mean age 76.8 ± 8.3 years; 74% males). All underwent complete diagnostic evaluation for suspected cardiac amyloidosis, including TTE with strain analysis, ECG, biomarkers, bone scintigraphy, and immunofixation.

**Figure 1:**
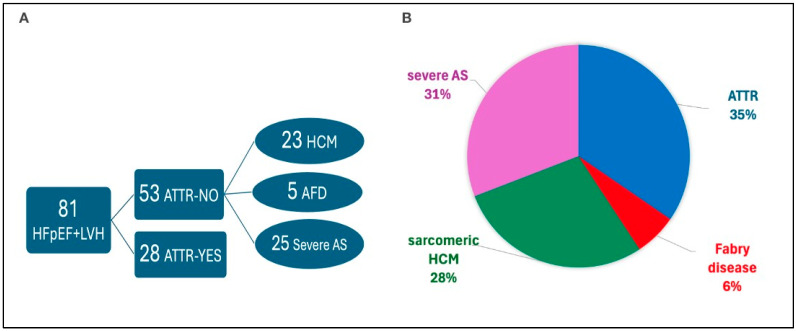
Final etiological classification of the 81 patients with HFpEF and LVH. (**A**) Diagnostic flow showing patients with confirmed or excluded ATTR. (**B**) Pie chart showing the relative proportions of ATTR, HCM, Fabry disease, and severe aortic stenosis (AS) among the cohort.

ATTR-CM was confirmed in 28 patients (34.5% of the cohort). Of these, 18 were ATTRwt and 10 were ATTRv. No patient had confirmed AL-CM, although one case of MGUS without end-organ involvement was identified. The remaining 53 patients had a hypertrophic phenotype of varying etiologies: these patients were diagnosed, after further examinations with sarcomeric hypertrophic cardiomyopathy (*n* = 23), Fabry disease (*n* = 5), and severe aortic stenosis (*n* = 24) (Figure 1).

**Figure 2:**
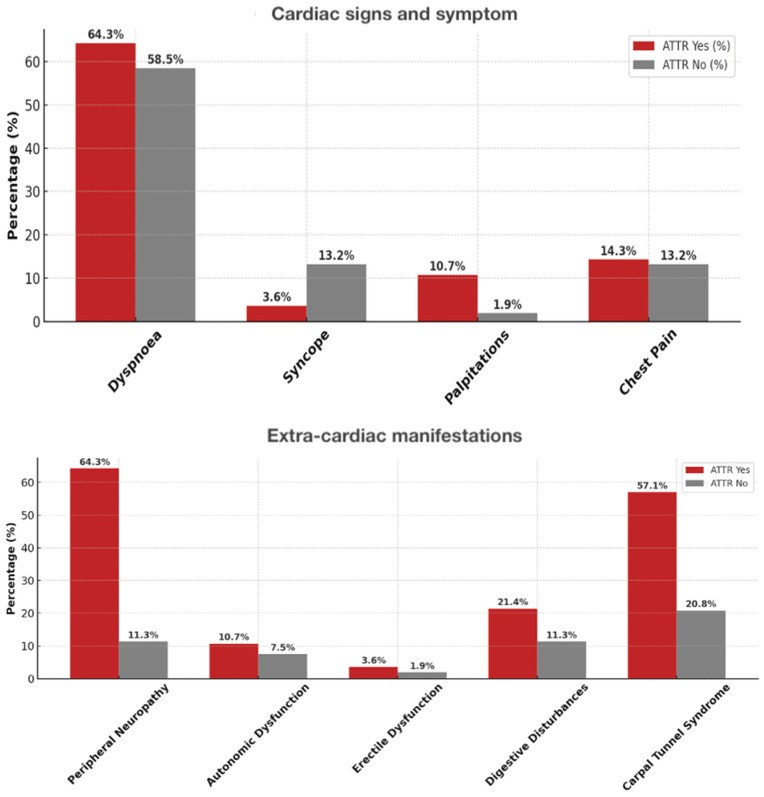

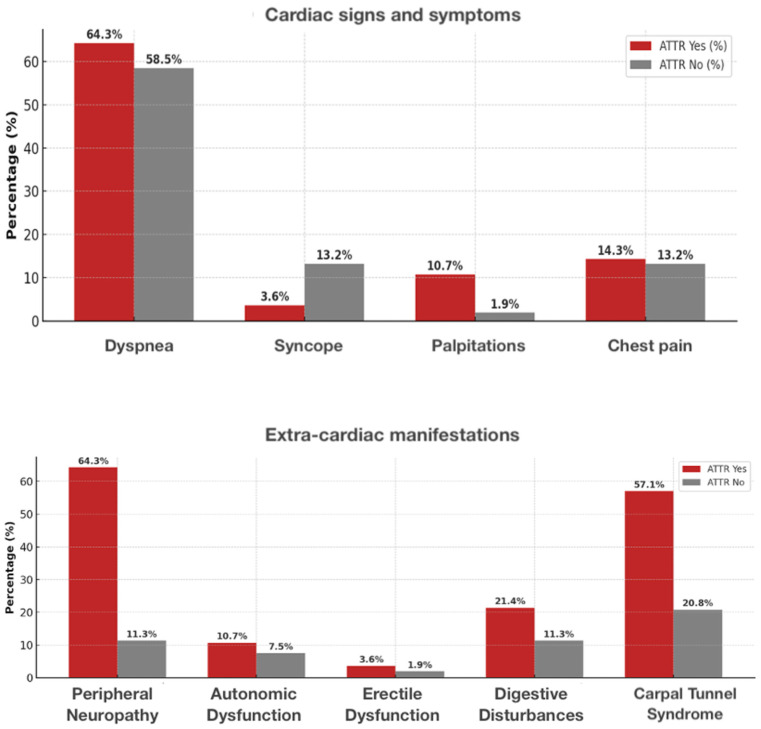
Prevalence of cardiac and extra-cardiac signs/symptoms in ATTR-positive vs. ATTR-negative patients. Upper panel—cardiac symptoms (dyspnea, syncope, palpitations, chest pain). Lower panel—extra-cardiac signs (peripheral neuropathy, autonomic dysfunction, erectile dysfunction, digestive disturbances, carpal tunnel syndrome).

Patients with ATTR-CM were older (80.6 ± 6.1 vs. 73.5 ± 8.4 years, *p* < 0.001) and more frequently male (88% vs. 62%, *p* = 0.013) than those without ATTR. In our cohort, cardiac symptoms such as exertional dyspnea and fatigue were common in both groups, but peripheral edema and signs of congestion were more frequent in ATTR CM patients (Figure 2).

Regarding extracardiac manifestations, bilateral carpal tunnel syndrome was significantly more prevalent in the ATTR CM group, often with onset years before cardiac symptoms. Lumbar spinal stenosis and biceps tendon rupture were also observed almost exclusively in ATTR CM, whereas peripheral neuropathy and autonomic symptoms were rare and showed no marked difference between groups (Figure 2). Further details are shown in Table 2 and Table 3.

In our cohort, the most frequently prescribed medications were diuretics, beta blockers, and ACE inhibitors/ARBs, with a higher use of loop diuretics in ATTR CM patients. Disease-modifying therapy with tafamidis was initiated exclusively in the ATTR CM group. Full details are reported in Table 2.

### 3.2. ECG and Echocardiographic Findings

On ECG, low QRS voltage was more frequent in ATTR-CM (41% vs. 15%, *p* = 0.017), as was grade I atrioventricular block (*p* = 0.007). There were no significant differences in arrhythmia prevalence between the groups (Table 4).

The mean interventricular septal thickness was significantly greater in ATTR CM patients compared with non ATTR (16.3 ± 2.1 mm vs. 13.2 ± 1.3 mm; *p* < 0.001), with a similar increase in posterior wall thickness. The relative wall thickness and left ventricular mass index were both markedly higher in the ATTR CM group, reflecting the concentric remodeling typical of amyloid infiltration. LV GLS was markedly reduced in ATTR CM patients compared with non-ATTR (*p* = 0.015), with a characteristic apical sparing pattern observed in 85% of ATTR CM cases versus 22.5% in non-ATTR (*p* < 0.001). LVEF was preserved in both groups, although slightly lower in ATTR CM (52.1% vs. 55.8%, *p* = 0.09).

Right ventricular impairment (TAPSE, RAA, RV diameter) and diastolic dysfunction were more marked in ATTR CM, with higher E/A (*p* = 0.020) and E/e′ (not significant), while LAVi was higher in the non-ATTR group. Pericardial effusion, when present, was more frequent in ATTR CM patients. Full echocardiographic data are presented in Table 4.

### 3.3. Biomarkers and Scintigraphy

The median NT-proBNP was higher in ATTR patients (2950 (1740–4730) pg/mL) than in non-ATTR (1340 (780–2480) pg/mL; *p* < 0.001). High-sensitivity troponin T was also higher in ATTR (35 (24–48) ng/L vs. 18 (12–26) ng/L; *p* < 0.001). All ATTR cases showed Grade 2 or 3 uptake on bone scintigraphy (Perugini Grade 3 in 68%), while all non-ATTR had Grade 0 or 1. Monoclonal protein testing was negative in all ATTR cases (Table 3).

### 3.4. Diagnostic Performance: T-Amylo vs. ATTR-CM (Davies)

- T-Amylo: sensitivity 91.2%, specificity 65.0%, PPV 70.8%, NPV 89.7%, overall accuracy 77.0%.
- Davies: sensitivity 73.5%, specificity 85.0%, PPV 80.5%, NPV 79.1%, overall accuracy 79.7%.

**Figure 3:**
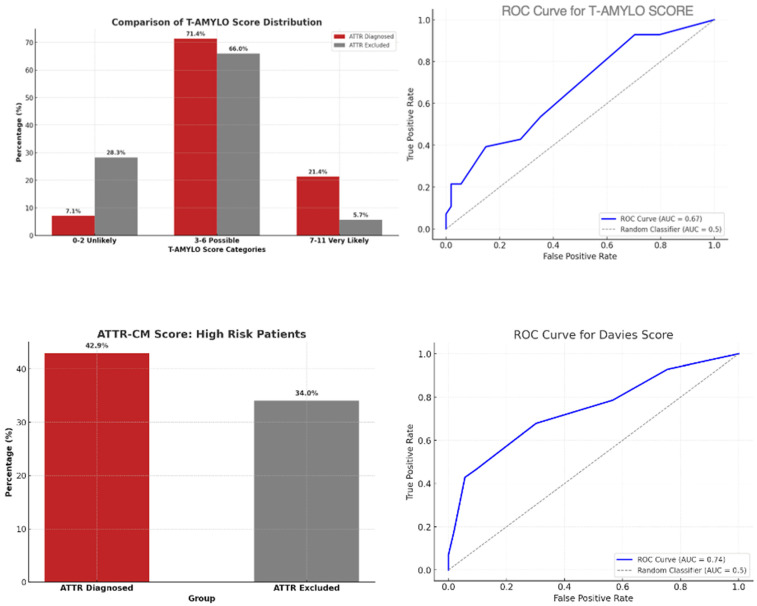
ATTR diagnostic scores and performance. (Upper left) T-AMYLO score distribution among patients with and without ATTR. (Upper right) ROC curve for T-AMYLO score (AUC = 0.67). (Lower left) ATTR-CM score: prevalence of high-risk patients in both groups. (Lower right) ROC curve for Davies score (AUC = 0.74).

ROC analysis showed an AUC of 0.87 (95% CI 0.79–0.95) for T-Amylo and 0.84 (95% CI 0.75–0.93) for Davies (*p* = 0.42 for AUC comparison) (Figure 3).

Agreement between the two scores was moderate (κ = 0.59). Both scores were positive in 62% of ATTR cases and negative in 55% of non-ATTR cases. Discordances occurred mainly in patients with borderline biomarker values or without apical sparing.

In summary:

- In the general population, T-Amylo was more sensitive, and Davies more specific.
- Agreement between T-Amylo and Davies was moderate, suggesting complementary diagnostic profiles.
- Dual-score strategies may optimize accuracy depending on the clinical setting.

## 4. Discussion

In this consecutive cohort of 81 patients undergoing full multimodal evaluation for suspected cardiac amyloidosis, we directly compared the diagnostic performance of T-Amylo and Davies scores.

In our cohort, key variables included in the T-Amylo and Davies scores clearly differentiated ATTR CM from non-ATTR patients. Bilateral carpal tunnel syndrome was markedly more frequent in ATTR CM, often preceding cardiac involvement. TTE showed greater interventricular septal thickness (16.3 ± 2.1 mm vs. 13.2 ± 1.3 mm) and apical sparing on LV GLS (85% vs. 22.5%). On ECG, low QRS voltage was observed in 41% of ATTR CM versus 15% of non-ATTR. Median NT proBNP was significantly higher in ATTR CM (2950 (1740–4730) pg/mL vs. 1340 (780–2480) pg/mL), and monoclonal protein testing was negative in all ATTR CM cases. These findings underscore the strong discriminatory power of the parameters underpinning both scores in our population.

Our data confirm that T-Amylo offers higher sensitivity (91.2%) and negative predictive value (89.7%), making it a valuable screening tool, while Davies provides greater specificity (85.0%) and positive predictive value (80.5%), supporting its role as a confirmatory instrument.

Agreement between the two tools was moderate (κ = 0.59), indicating that they identify overlapping but not identical patient subsets.

### 4.1. Comparison with Existing Literature

The T-Amylo score showed strong discriminatory power (AUC ≈ 0.86) in 227 patients with suspected CA (derivation cohort): it has been validated in a sample of 895 patients from 11 centers across various cardiology settings, including hypertensive cardiopathy, severe aortic stenosis, and HFpEF, showing a good performance (AUC 0.82) [18].

Recent prospective work by Burgos et al. (2025) in acute heart failure patients confirmed excellent diagnostic accuracy (AUC 0.93), with 100% sensitivity in low-risk groups and specificity > 97% in high-risk groups—findings consistent with our NPV and overall sensitivity [23].

The Davies (ATTR CM) score, derived and validated in HFpEF populations by Davies et al. [19], achieved AUCs of 0.84–0.89 and high specificity (~85%) in HFpEF populations. Our specificity result matches these reports, confirming that the Davies score is particularly robust for ruling in ATTR CM when positive.

Our findings can also be compared with a recent single-center study conducted in a highly diverse U.S. cohort (*n* = 476; 65% non-Hispanic Black, 20% Hispanic, 45% female) in which the T-Amylo score demonstrated lower discriminatory power (AUC 0.75) compared with the ATTR CM (Davies) score (AUC 0.86, *p* < 0.001) and a locally derived multivariate model (AUC 0.92) [24]. In our predominantly Caucasian population with a high prevalence of ATTRwt, T-Amylo maintained excellent sensitivity (91.2%) and overall accuracy (AUC 0.87), consistent with prior European validations. The reduced performance observed in the U.S. study may reflect differences in phenotype distribution, genetic background (e.g., higher prevalence of the V122I variant in Black patients), and the proportion of female patients, all of which may influence the predictive value of clinical variables incorporated in the score. These observations underscore the importance of validating diagnostic scores across diverse populations and adapting them to local epidemiology when integrating them into screening algorithms.

### 4.2. Clinical Implications

In daily practice, T-Amylo may be best suited as a first-line screening tool in patients with unexplained LVH or HFpEF, due to its ability to minimize false negatives.

Its combination of simple clinical, echocardiographic, and biomarker parameters makes it widely applicable, including in centers without immediate access to advanced imaging.

The Davies score is better suited for diagnostic confirmation in high-probability cases, particularly when scintigraphy access is limited or when clinicians aim to prioritize specificity before initiating costly or long-term therapies.

Given the moderate concordance between the two scores, a two-step strategy could be adopted:

- Apply T-Amylo to screen and select patients for further evaluation;
- Use Davies to confirm high-probability cases before advanced imaging or biopsy.

This approach could optimize resource utilization and reduce unnecessary testing.

### 4.3. Alignment with Evolving Diagnostic Frameworks

The diagnostic approach to ATTR CM continues to evolve with updated consensus recommendations [1,8,9] and standardization of scintigraphy protocols. Simultaneously, old and new disease-modifying therapies such as TTR stabilizers and emerging agents like gene silencers, gene-editing (CRISPR-Cas9 technology) therapies, and antibody-mediated amyloid removal make the early identification of ATTRwt crucial. In this therapeutic context, efficient triage systems using T-Amylo and Davies could shorten the time from suspicion to treatment initiation [9].

### 4.4. Limitations and Future Directions

Our study is limited by its retrospective, single-center design and relatively small sample size, which may reduce generalizability. The predominance of elderly male patients reflects known ATTRwt epidemiology but limits the evaluation of sex-specific performance. We did not evaluate in the present study prognostic outcomes or treatment responses according to baseline score classification, which remain important questions for future research. A potential limitation of our study is the selection bias inherent to our cohort, as all patients were referred for suspected cardiac amyloidosis and therefore had a relatively high prevalence of confirmed ATTR. This context may have increased the sensitivity of both T-Amylo and Davies scores compared with what could be expected in an unselected heart failure population, where the pre-test probability of ATTR is lower. Consequently, caution is warranted when extrapolating our results to general HFpEF cohorts.

Future work should include

- Prospective multicenter validation in more diverse populations (including women and non-Caucasian patients).
- Integration with advanced imaging (CMR extracellular volume, PET tracers) to refine intermediate-risk classification.
- Automated calculation of scores within electronic health records to trigger early referral.
- Prognostic studies to determine whether baseline score levels predict response to tafamidis or RNA silencing therapies.

An interesting future perspective may be the development of a novel score that integrates the most sensitive and specific variables from both T-Amylo and Davies. Such a tool could be particularly useful for patients who cannot undergo advanced diagnostic modalities such as scintigraphy, cardiac MRI, or biopsy. Our study may serve as a preliminary step in identifying which features should be prioritized in the design of such a combined score, although its formal development and validation would require larger, prospective datasets.

Recent advances in artificial intelligence provide new opportunities to enhance diagnostic accuracy, manage large and complex datasets, and improve longitudinal disease monitoring. Automated algorithms applied to routine ECGs and echocardiograms—such as those developed by Mayo Clinic investigators [25,26]—may facilitate earlier identification of cardiac amyloidosis and enable more timely initiation of disease-modifying therapies. As machine learning approaches continue to evolve, their integration with validated clinical scores such as T-Amylo and Davies could support the development of highly accurate, fully automated diagnostic pathways for ATTR CM.

## 5. Conclusions

In a real-world cohort of patients with suspected cardiac amyloidosis, the T-Amylo and Davies scores demonstrated complementary diagnostic profiles. T-Amylo provided high sensitivity and negative predictive value, supporting its role as a screening tool to minimize missed diagnoses. Davies showed superior specificity and positive predictive value, confirming its utility for ruling in disease in high-probability cases. Moderate concordance between scores highlights their ability to capture distinct aspects of the ATTR CM phenotype. A sequential approach—screening with T-Amylo and confirming with Davies—may optimize diagnostic pathways, streamline resource use, and accelerate access to disease-modifying therapies in this increasingly treatable condition.

Highlights:

- ATTRwt-CM is underdiagnosed in elderly patients with HFpEF and LVH.
- We compared T-Amylo and Davies scores in a consecutive real-world cohort.
- T-Amylo: sensitivity 91.2%, high NPV—ideal for initial screening.
- Davies: specificity 85.0%, high PPV—ideal for confirmation.
- Moderate agreement suggests complementary diagnostic roles.
- Sequential use of T-Amylo followed by Davies may optimize efficiency. This approach could reduce unnecessary imaging and expedite therapy.

## Figures and Tables

**Table 1:** Diagnostic scores’ calculation, adapted from the original articles.

T-AMYLO Score	
Age ≥ 80 ys	1 pt
IVSd thickness ≥ 16 mm	2 pt
Low QRS voltage	2 pt
Male gender	3 pt
Carpal tunnel syndrome	3 pt
High risk: 7–11 pt	
Bone scintigraphy and light chain analysis	
Intermediate risk: 3–6 pt	
Reconsider red flags	
Low risk: 0–2 pt	
Reconsider differential diagnosis of hypertrophic phenotype	
Adapted from [18].	
**ATTR-CM Score**	
Age	
60–69	+2 pt
70–79	+3 pt
≥80	+4 pt
Male gender	+2 pt
Hypertension diagnosis	−1 pt
Ejection fraction (EF) < 60%	+1 pt
PWd thickness ≥ 12 mm	+1 pt
Relative wall thickness > 0.57	+2 pt
High risk score ≥ 6 Require confirmatory bone scintigraphy	
Adapted from [19].	

**Table 2:** General characteristics of study population.

Parameter	ALL N = 81	ATTR Yes N = 28	ATTR No N = 53	*p*-Value
ATTR wild-type, *n* (%)	18 (22.2)	18 (64.3)	0 (0)	**<0.001**
ATTR variant, *n* (%)	10 (12.3)	10 (35.7)	0 (0)	**<0.001**
Age, years	71.8 ± 12.7	69.2 ± 16.8	73.2 ± 9.8	0.176
M, *n* (%) F, *n* (%)	49 (60.5) 32 (39.5)	19 (67.9) 9 (32.1)	30 (56.6) 23 (43.4)	**0.032**
BSA, mq	1.8 ± 0.26	1.8 ± 0.26	1.8 ± 0.26	0.884
BMI, kg/mq	27.3 ± 5.3	28.8 ± 4.4	27.6 ± 5.7	0.533
NYHA 1, *n* (%)	9 (11.1)	7 (25.0)	2 (3.8)	**0.003**
NYHA 2, *n* (%)	56 (69.1)	18 (64.3)	38 (71.7)	0.613
NYHA 3, *n* (%)	16 (19.8)	3 (10.7)	13 (24.5)	0.137
HR, bpm	68.6 ± 11.0	67.3 ± 11.8	69.3 ± 10.5	0.438
BPs, mmHg	132.4 ± 15.1	132.1 ± 13.4	132.5 ± 16.1	0.928
BPd, mmHg	76.7 ± 10.3	79.8 ± 6.6	74.9 ± 11.5	**0.043**
Hypertension, *n* (%)	66 (81.5)	22 (78.6)	44 (83)	0.624
Diabetes, *n* (%)	17 (21.0)	4 (14.3)	13 (24.5)	0.281
Dyslipidemia, *n* (%)	42 (51.9)	12 (42.9)	30 (56.6)	0.239
Pharmacological therapy				
Beta-blockers, *n* (%)	52 (64.2)	14 (50)	38 (71.7)	0.052
MRAs	10 (12.3)	7 (25)	3 (5.7)	**0.012**
Amyodarone	3 (3.7)	0 (0)	3 (5.7)	0.199
Other AAD	1 (1.2)	1 (3.6)	0 (0)	0.166
ACEi/ARBs	47 (58.0)	15 (53.6)	32 (60.4)	0.555
Diuretics	43 (53.1)	15 (53.6)	28 (52.8)	0.949
Anticoagulants	22 (27.2)	9 (32.1)	13 (24.5)	0.463
Antiplatelets	34 (42.0)	6 (21.4)	28 (52.8)	**0.006**

Note: Statistically significant *p*-values (*p* < 0.05) are highlighted in bold. Abbreviations: ATTR (amyloid transthyretin); F (females); M (males); BSA (body surface area); BMI (body mass index); NYHA (New York Heart Association); HR (heart rate); BPd (diastolic blood pressure); BPs (systolic blood pressure); MRA (mineralcorticoid receptor antagonist); ACEi (angiotensin-converting enzyme inhibitors); ARBs (angiotensin II receptor blockers).

**Table 3:** Symptoms, signs, and biomarkers; extracardiac manifestations.

	ALL N = 81	ATTR Yes N = 28	ATTR No N = 53	*p*-Value
Dyspnea (*n*, %)	49 (60.5)	18 (64.3)	31 (58.5)	0.641
Syncope	8 (9.9)	1 (3.6)	7 (13.2)	0.166
Palpitations	4 (4.9)	3 (10.7)	1 (1.9)	0.081
Chest pain	11 (13.6)	4 (14.3)	7 (13.2)	0.892
CAD	9 (11.1)	0 (0)	9 (17.0)	**0.020**
PCI	4 (4.9)	0 (0)	4 (7.5)	0.136
TAVR	25 (30.9)	0 (0)	25 (47.2)	**<0.001**
Scintigraphy done	19 (23.5)	18 (64.3)	1 (1.9)	**<0.001**
Perugini score				
1, *n* (%)	0 (0)	0 (0)		
2, *n* (%)	8 (9.9)	8 (28.6)		
3, *n* (%)	9 (11.1)	9 (32.1)		
BNP, pg/mL	210 (99; 465)	349 (100; 457)	193 (96; 490)	0.667
NT-proBNP, pg/mL	2225 (733; 3903)	2226 (733; 3903)		
Troponin, ng/mL	25.5 (12.8; 49.8)	40 (22; 71)	19 (10; 30)	0.060
Extracardiac manifestations				
Peripheral neuropathy, *n* (%)	24 (29.6)	18 (64.3)	6 (11.3)	**<0.001**
Autonomic dysfunction	7 (8.6)	3 (10.7)	4 (7.5)	0.629
Erectile dysfunction	2 (2.5)	1 (3.6)	1 (1.9)	0.642
Incontinence	8 (9.9)	0 (0)	8 (15.1)	**0.030**
Digestive disturbances	12 (14.8)	6 (21.4)	6 (11.3)	0.223
Itch	4 (4.9)	0 (0)	4 (7.5)	0.136
Carpal tunnel syndrome	27 (33.3)	16 (57.1)	11 (20.8)	**0.001**
Lumbar stenosis	3 (3.7)	3 (10.7)	0 (0)	**0.015**
Creatinine, mg/dl	1 (0.9; 1.3)	1.1 (0.9; 1.3)	1.0 (0.9; 1.7)	0.874
eGFR, ml/min/mq	59 ± 25	69 ± 21	53 ± 26	**0.046**

Note: Statistically significant *p*-values (*p* < 0.05) are highlighted in bold. Abbreviations: CAD (coronary artery disease); eGFR (estimated glomerular filtration rate); PCI (percutaneous coronary intervention); TAVR (transcatheter aortic valve replacement); BNP (B-type natriuretic peptide); NT-proBNP (N-terminal pro B-type natriuretic peptide).

**Table 4:** ECG and transthoracic echo data.

Parameter	ALL N = 81	ATTR Yes N = 28	ATTR No N = 53	*p*-Value
ECG and arrhythmias				
Holter done, *n* (%)	24 (29.6)	9 (32.1)	15 (28.3)	0.718
NSVT	4 (4.9)	1 (3.6)	3 (5.7)	0.679
AF	23 (28.4)	6 (21.4)	17 (32.1)	0.312
PR duration, ms	179.9 ± 42.7	194.6 ± 52.6	172.5 ± 35.3	0.058
QRS duration, ms	115.1 ± 25.5	113.1 ± 26.1	115.7 ± 25.6	0.742
AVB I, *n* (%)	18 (22.2)	11 (39.3)	7 (13.2)	**0.007**
AVB III	2 (2.5)	1 (3.6)	1 (1.9)	0.642
LBBB	5 (6.2)	2 (7.1)	3 (5.7)	0.792
RBBB	21 (25.9)	6 (21.4)	15 (28.3)	0.502
LAFB	3 (3.7)	2 (7.1)	1 (1.9)	0.233
Low QRS voltage	6 (7.4)	5 (17.9)	1 (1.9)	**0.009**
Pseudo-infarction pattern	5 (6.2)	2 (7.1)	3 (5.7)	0.823
PM implantation	8 (9.9)	3 (10.7)	5 (9.4)	0.854
ICD implantation	3 (3.7)	0 (0)	3 (5.7)	0.199
Echo parameters				
IVSd, mm	14.0 ± 3.1	13.2 ± 3.2	14.5 ± 3.0	0.095
LVPWd, mm	12.0 ± 2.7	12.3 ± 2.6	11.8 ± 2.8	0.507
LVMi, g/mq	140.7 ± 40.5	136.8 ± 40.4	144.6 ± 41.0	0.483
RWT	0.51 ± 0.16	0.51 ± 0.18	0.51 ± 0.15	0.942
EF, %	60 ± 7	60 ± 4	60 ± 8	0.537
E/A	1.1 (0.82; 1.78)	1.4 (0.97; 2.02)	1.0 (0.66; 1.22)	**0.020**
E/e′	13.2 (11.1; 18.5)	14.9 (11.5; 18.6)	12.5 (10.1; 18.0)	0.244
TRVmax, m/s	2.7 ± 0.5	2.7 ± 0.5	2.6 ± 0.6	0.601
LAVi, ml/mq	41.8 ± 17.0	35.5 ± 14.8	45.8 ± 17.4	**0.015**
RVIDd, cm	32.3 ± 4.45	34 ± 2.5	31.8 ± 4.7	0.220
RAA, ml/mq	21.13 ± 7.2	22.12 ± 6.8	20.5 ± 7.6	0.606
TAPSE, mm	2.1 (1.8; 2.3)	1.8 (1.6; 2.0)	2.2 (1.9; 2.4)	**<0.001**
LV-GLS, %	−15 (−10; −19)	−13 (−10; −17)	−17.5 (−12.3; −19.7)	**0.004**
Apical sparing, *n* (%)	16 (19.8)	15 (53.6)	1 (1.9)	**<0.001**
Pericardial effusion, *n* (%)	21 (25.9)	11 (39.3)	10 (18.9)	**0.046**
Aortic stenosis, *n* (%)	30 (37.0)	3 (10.7)	27 (50.9)	**<0.0001**
Severe, *n* (%)	25 (30.9)	0 (0)	25 (47.2)	**<0.001**

Note: Statistically significant *p*-values (*p* < 0.05) are highlighted in bold. Abbreviations: NSTV (non-sustained ventricular tachycardia); STV (sustained ventricular tachycardia); PVBs (premature ventricular beats); AF (atrial fibrillation); AVB I (first-degree atrioventricular block); AVB III (third-degree atrioventricular block); LBBB (left bundle branch block); RBBB (right bundle branch block); LAFB (left anterior fascicular block); PM (pacemaker); ICD (implantable cardioverter defibrillator).

## Data Availability

The data underlying this article are not available in a publicly accessible repository due to technical constraints and privacy considerations. They will be shared on reasonable request to the corresponding author, in fully anonymized form and in compliance with ethical and legal requirements.

## Supplementary Materials

The STROBE Checklist for observational studies can be downloaded at: https://www.mdpi.com/article/10.3390/jcdd12110417/s1.

## Abbreviations

The following abbreviations are used in this manuscript:

CA	Cardiac Amyloidosis.
HFpEF	Heart Failure with Preserved Ejection Fraction.
LVH	Left Ventricular Hypertrophy.
ATTRwt(-CM)	Wild-Type Transthyretin Cardiac Amyloidosis.
ATTRv	Variant Transthyretin Amyloidosis.
ATTR-CM	Transthyretin Cardiac Amyloidosis (generico).
AL-CM	Light-chain Cardiac Amyloidosis.
ECG	Electrocardiogram.
TTE	Transthoracic Echocardiography.
NT-proBNP	N-Terminal Pro-B-type Natriuretic Peptide.
GLS	Global Longitudinal Strain.
AUC	Area Under the Curve.
PPV	Positive Predictive Value.
NPV	Negative Predictive Value.
