# Supplementary material for: Diagnostic Predictive Scores of Amyloid Cardiomyopathy in Patients with Heart Failure with Preserved Ejection Fraction and Left Ventricular Hypertrophy
Source: J Cardiovasc Dev Dis. 2025 Oct 22;12(11):417. doi: 10.3390/jcdd12110417 (PMC12653825; doi:10.3390/jcdd12110417)
Supplement: Supplementary file 1 [file jcdd-12-00417-s001.zip › jcdd-3908669-supplementary.pdf]

STROBE Statement—checklist of items that should be included in reports of observational studies

Title: Diagnostic predictive scores of Amyloid Cardiomyopathy in patients with Heart Failure with Preserved Ejection Fraction and Left Ventricular Hypertrophy

Authors: Denise Cristiana Faro, Fabrizia Romeo, Valentina Losi, Dario Simonetti, Davide Capodanno, Ines Paola Monte

	Item No	Recommendation
**Title and abstract**	1	*(a)* Indicate the study's design with a commonly used term in the title or the abstract<br>Reported/Addressed, Structured Abstract, Design stated as "retrospective observational" and that 81 consecutive patients were analyzed.
		(*b*) Provide in the abstract an informative and balanced summary of what was done and what was found<br>Reported/Addressed, Background, Objectives, Methods, Results (incl. accuracy metrics), and Conclusions reported.
**Introduction**		
Background/rationale	2	Explain the scientific background and rationale for the investigation being reported<br>Reported/Addressed, Introduction (first 4 paragraphs): HFpEF/LVH underdiagnosis of ATTRwt-CM, importance of early noninvasive diagnosis; rationale for comparing TAmylo vs Davies
Objectives	3	State specific objectives, including any prespecified hypotheses<br>Reported/Addressed, Introduction (last paragraph): Objective = compare diagnostic performance of TAmylo and Davies scores in a real-world cohort; hypothesis = complementary roles.
**Methods**		
Study design	4	Present key elements of study design early in the paper<br>Reported/Addressed, Methods §2.1 Study Design and Population<br>Retrospective observational, consecutive series at a tertiary referral center, cross-sectional (no follow-up study)
Setting	5	Describe the setting, locations, and relevant dates, including periods of recruitment, exposure, follow-up, and data collection<br>Reported/Addressed, Methods §2.1: Tertiary referral center; inclusion window Jan 2021–Jun 2023; cross-sectional diagnostic assessment (no longitudinal follow-up).
Participants	6	(*a*) *Cohort study*—Give the eligibility criteria, and the sources and methods of selection of participants. Describe methods of follow-up<br>*Cross-sectional study*—Give the eligibility criteria, and the sources and methods of selection of participants<br>Reported/Addressed: Methods §2.1: Inclusion = age ≥18, LV wall thickness ≥12 mm, LVEF >50%, HF signs/symptoms, complete work-up (ECG, TTE+strain, NT-proBNP/hs-Tn, bone scintigraphy, immunofixation/FLC). Exclusions: incomplete data, poor echo window, non-collaborative, prior CA diagnosis, prior disease-modifying therapy.
		(*b*) *Cohort study*—For matched studies, give matching criteria and number of exposed and unexposed<br>N/A (not a matched study).

Variables	7	Clearly define all outcomes, exposures, predictors, potential confounders, and effect modifiers. Give diagnostic criteria, if applicable<br>Reported/Addressed, Methods §2.2–2.2.3: Outcome = ATTR-CM diagnosis (non-invasive criteria according to guidelines). Predictors: variables comprising TAmylo and Davies; thresholds prespecified. Potential confounders minimized by inclusion/exclusion
Data sources/ measurement	8*	For each variable of interest, give sources of data and details of methods of assessment (measurement). Describe comparability of assessment methods if there is more than one group<br>Reported/Addressed, Methods §2.2.1–2.2.3: ECG (standard 12-lead; low voltage, pseudo-infarct); TTE (Vivid E95; GLS with apical sparing definition); Bone scintigraphy (99mTc-DPD/HMDP; Perugini grading); Immunofixation/FLC; Biomarkers (NT-proBNP, hs-Tn). Same protocols across groups.
Bias	9	Describe any efforts to address potential sources of bias<br>Reported/Addressed, Methods §2.2.1–§2.2.3; §2.1 Consecutive sampling; standardized diagnostic protocol aligned with guidelines; multiple imaging readers; exclusion of incomplete datasets; clear reference standard for ATTR-CM
Study size	10	Explain how the study size was arrived at<br>Reported/Addressed, Methods §2.1; Results §3.1: Convenience sample of all consecutive eligible patients within the timeframe (n=81). No a priori power calculation
Quantitative variables	11	Explain how quantitative variables were handled in the analyses. If applicable, describe which groupings were chosen and why<br>Reported/Addressed, Methods §2.3: Mean±SD or median[IQR]; prespecified thresholds for scores (TAmylo≥4; Davies≥3–4 per original definitions.).
Statistical methods	12	*(a)* Describe all statistical methods, including those used to control for confounding<br>Reported/Addressed, Methods §2.3. Sensitivity, specificity, PPV, NPV, accuracy; Cohen's κ for agreement; ROC/AUC with DeLong comparison. No multivariable adjustment (diagnostic accuracy focus).
		(b) Describe any methods used to examine subgroups and interactions<br>Reported/Addressed, Results §3.4: Discordant-case analysis; agreement assessment; no formal interaction modelling
		(c) Explain how missing data were addressed<br>Reported/Addressed, Methods §2.1; Results §3.1: Excluded incomplete cases a priori; analytic dataset complete.
		(*d*) *Cohort study*—If applicable, explain how loss to follow-up was addressed<br>*Case-control study*—If applicable, explain how matching of cases and controls was addressed<br>*Cross-sectional study*—If applicable, describe analytical methods taking account of sampling strategy<br>Reported/Addressed, Methods §2.1: Cross-sectional consecutive sampling of referrals; no weighting.
		(*e*) Describe any sensitivity analyses<br>Not performed

Continued on next page

**Results**		
Participants	13*	*(a)* Report numbers of individuals at each stage of study—eg numbers potentially eligible, examined for eligibility, confirmed eligible, included in the study, completing follow-up, and analysed<br>Reported/Addressed, Results §3.1; Figure 1: 81 included; 28 ATTR-CM; 53 non-ATTR.
		(b) Give reasons for non-participation at each stage<br>Reported/Addressed, Methods §2.1; Figure 1: Exclusions for incomplete data, poor echo, prior diagnosis or therapy.
		(c) Consider use of a flow diagram<br>Reported/Addressed: Figure 1.
Descriptive data	14*	*(a)* Give characteristics of study participants (eg demographic, clinical, social) and information on exposures and potential confounders<br>Reported/Addressed, Results §3.1–3.3; Tables 2–4; Figure 2. Demographics/clinical; ECG/TTE; biomarkers; extracardiac red flags; medication use
		(b) Indicate number of participants with missing data for each variable of interest<br>Reported/Addressed, Methods §2.1. Core diagnostic variables complete in analytic set; indicate '0' or 'NA' per table footnotes if requested
		(c) *Cohort study*—Summarise follow-up time (eg, average and total amount)<br>N/A (cross-sectional diagnostic study).
Outcome data	15*	*Cohort study*—Report numbers of outcome events or summary measures over time
		Case-control study—Report numbers in each exposure category, or summary measures of exposure
		Cross-sectional study—Report numbers of outcome events or summary measures<br>Reported/Addressed, Results §3.4; Figure 3: *ATTR-CM confirmed 28/81 (34.5%); diagnostic metrics reported; ROC/AUC for both scores.*
Main results	16	(*a*) Give unadjusted estimates and, if applicable, confounder-adjusted estimates and their precision (eg, 95% confidence interval). Make clear which confounders were adjusted for and why they were included<br>Reported/Addressed, Results §3.4; Figure 3: diagnostic metrics; AUC with 95% CI.
		(b) Report category boundaries when continuous variables were categorized<br>Reported/Addressed, Methods §2.2.3: Score thresholds prespecified (TAmylo ≥4; Davies ≥3–4).
		(a) If relevant, consider translating estimates of relative risk into absolute risk for a meaningful time period<br>Not applicable.
Other analyses	17	Report other analyses done—eg analyses of subgroups and interactions, and sensitivity analyses<br>Reported/Addressed, Results §3.4; Discussion §4.2–4.3: agreement (κ=0.59); discordant cases.
**Discussion**		
Key results	18	Summarise key results with reference to study objectives<br>Reported/Addressed, Discussion §4.0: TAmylo higher sensitivity/NPV; Davies higher specificity/PPV; sequential use suggested.
Limitations	19	Discuss limitations of the study, taking into account sources of potential bias or imprecision. Discuss both direction and magnitude of any potential bias<br>Reported/Addressed, Discussion §4.4: retrospective, single-center, small cohort, elderly male predominance, no prognostic follow-up.
Interpretation	20	Give a cautious overall interpretation of results considering objectives, limitations, multiplicity

		of analyses, results from similar studies, and other relevant evidence<br>Reported/Addressed, Discussion §4.1–§4.3: consistent with previous validations; complementary use; clinical implications for practice and evolving frameworks discussed.
Generalisability	21	Discuss the generalisability (external validity) of the study results<br>Reported/Addressed, Discussion §4.4: Applicability to similar HFpEF/LVH populations; need for multicenter and diverse cohorts.
**Other information**		
Funding	22	Give the source of funding and the role of the funders for the present study and, if applicable, for the original study on which the present article is based<br>Reported/Addressed, Acknowledgements/Funding: No external funding; institutional only.

*Give information separately for cases and controls in case-control studies and, if applicable, for exposed and unexposed groups in cohort and cross-sectional studies.

Note: An Explanation and Elaboration article discusses each checklist item and gives methodological background and published examples of transparent reporting. The STROBE checklist is best used in conjunction with this article (freely available on the Web sites of PLoS Medicine at http://www.plosmedicine.org/, Annals of Internal Medicine at http://www.annals.org/, and Epidemiology at http://www.epidem.com/). Information on the STROBE Initiative is available at www.strobe-statement.org.
